# Supplementary material for: MetaRibo-Seq measures translation in microbiomes
Source: Nat Commun. 2020 Jun 29;11:3268. doi: 10.1038/s41467-020-17081-z (PMC7324362; doi:10.1038/s41467-020-17081-z)
Supplement: Supplementary file 10 — Supplementary Data 7 [file 41467_2020_17081_MOESM10_ESM.zip › File2/Confidence_High_Taxonomy/204025_out.krona.html]

Javascript must be enabled to view this page.

members
magnitude
magnitudeUnassigned
count
unassigned
taxon
rank

204025\_out

2

superkingdom
2
2

phylum
2
1239

class
2
186801

2
186802
order

family
186803
1

841
1
genus

species

SRS047014\_contig\_number\_314
1
2049040

541000
1
family

1263
1
genus


SRS015854\_contig\_number\_contig-100\_4513.107533
species
165186
1
